# How many strides are required for a reliable estimation of temporal gait parameters? Implementation of a new algorithm on the phase coordination index

**DOI:** 10.1371/journal.pone.0192049

**Published:** 2018-02-08

**Authors:** Lotem Kribus-Shmiel, Gabi Zeilig, Boris Sokolovski, Meir Plotnik

**Affiliations:** 1 Center of Advanced Technologies in Rehabilitation, Sheba Medical Center, Tel Hashomer, Israel; 2 Sackler School of Medicine, Tel-Aviv University, Tel Aviv, Israel; 3 Department of Neurological Rehabilitation, Sheba Medical Center, Tel Hashomer, Ramat Gan, Israel; 4 Department of Physiology and Pharmacology, Sackler School of Medicine, Tel-Aviv University, Tel Aviv, Israel; 5 Sagol School of Neuroscience, Tel-Aviv University, Tel Aviv, Israel; Universitat zu Koln, GERMANY

## Abstract

**Background:**

The Phase coordination index (PCI), a temporal gait measure that quantifies consistency and accuracy in generating the anti-phased left-right stepping pattern, assesses bilateral coordination of gait in various cohorts (e.g., Parkinson's disease, post stroke). As PCI is based on mean values calculated across a series of gait cycles, individuals are required to perform lengthy walking trials, prolonging gait assessments which cause discomfort to some of them. This study introduces an algorithm to identify the required number of strides to obtain a reliable, characteristic PCI value.

**Methods:**

Simulated data sets, as well as physiological data (obtained from healthy elderly and young persons, from over ground and treadmill trials) were used in this research. A series of N-1 PCI values was calculated for i = 2,3,4…N gait cycles for each participant. There is a value i = k, representing certain number of cycles, for which no significant change in PCI occurs as additional cycles are added, termed point of stabilization (POS). The algorithm presented here uses a 2-stage iterative process to determine POS. Stage 1 searches for the gross location of the interval of PCI values containing the POS. In stage 2, the algorithm performs a high-resolution recursive, iterative process within this interval to find the exact point. The criterion for defining stability within a window of PCI values is a coefficient of variation (CV) of ≤ 5%.

**Results:**

Our recursive, iterative algorithm indicates that ~23 strides on average should be captured to attain a characteristic PCI.

**Conclusions:**

Gait trials with at least 23 strides on average should suffice to obtain a reliable estimation of PCI in healthy young adults. While this methodology may be considered generic, future studies should obtain POS values based on additional cohorts (e.g., disabled participants, fixed walking speeds).

## Introduction

### The problem—How many gait cycles are needed to characterize temporal gait parameters?

In recent years, there has been increasing interest in gait analysis of temporal gait parameters and their long term dynamics. Therefore, in some experiments, gait measurements are obtained during relatively long walking periods, with the use of treadmills (TMs) [1] and also with wearable gait technologies during over ground walking [1–6]. Thus, data from a large number of gait cycles can be collected, avoiding the limitations imposed by the confined space of classic gait analysis laboratories [7].

In order to assess gait variability, the coefficient of variation (CV) of the stride-to-stride-time [5, 8] is calculated. To assess bilateral coordination of gait, the phase coordination index (PCI), can be calculated [9]. The PCI quantifies the degree of the consistency and the accuracy in generating anti phased left-right stepping [10–13]. This article focuses on the PCI measurement.

For calculating PCI, vectors of left right stepping phase values, φ, are calculated from the timing of consecutive heel strikes. Mean values are calculated, which requires a sufficient number of gait cycles. Thus, on one hand experimental gait trials should be long enough to obtain a ‘sufficient’ number of gait cycles, but, on the other hand, lengthy trials can cause fatigue in the elderly and in participants with maladies, and consequently may affect the sampling [14].

The objective of this theoretical assay is to estimate the required number of gait cycles which is sufficient to reliably assess the value of the PCI in a gait trial. In other words, to identify from which gait cycle and on, additional data will not yield a significant change in the value of PCI.

### Stabilization period detection methods

Detection of a stabilization period (steady-state) in any series of data (or signal) is important for performance assessment [15, 16]. Steady state refers to the state where the mean of a series is practically unchanging, thus represents the true nature of a system. At present, many offline detection methods exist [17–19]. Examples for such methods include cumulative sum (CUSUM) plots [20], exponentially weighted moving average control charts [21], statistical process control method[19] and the marginal standard error rules (MSER and MSER-5) [22, 23]. Other methods include the goodness-of-fit test [24], wavelet-based spectral method [25, 26], batch-means-based tests [27], t-tests, F-tests [28], the sequential method [24], the scale invariant truncation point method [29] and filter method [30]. These offline steady-state detection methods have some downsides that should be acknowledged: (a) relatively lengthy calculations (e.g., [22, 31]); (b) statistical tests are sensitive to the distribution of the test statistics, making it challenging to modify the algorithm once implemented in a software application; and (c) may be complex (e.g., wavelet transform method,[26]).

Additionally, a few online detection methods exist and include, for example (a) slope detection method (SDM) where linear regression is performed over a moving window and the fitted slope is tested [32–34]; (b) t-test on two means of two adjacent windows with pooled standard-deviation [35]; (c) variances ratio test [36]; (d) algorithm using Bayesian inference techniques [31]. The online methods have certain limitations as well, e.g., one common disadvantage is that a data window must be used. A moving window that is too wide may spuriously delay the detection of steady state, while too narrow a window may increase the false detection rate.

This paper proposes a steady-state detection algorithm based on size-adapting moving windows and calculating the coefficient of variation (CV = standard deviation/mean). This algorithm was tested for usability and feasibility on simulated gait data and was shown applicable on real data.

## Methods

### PCI definition

**Fig 1:**
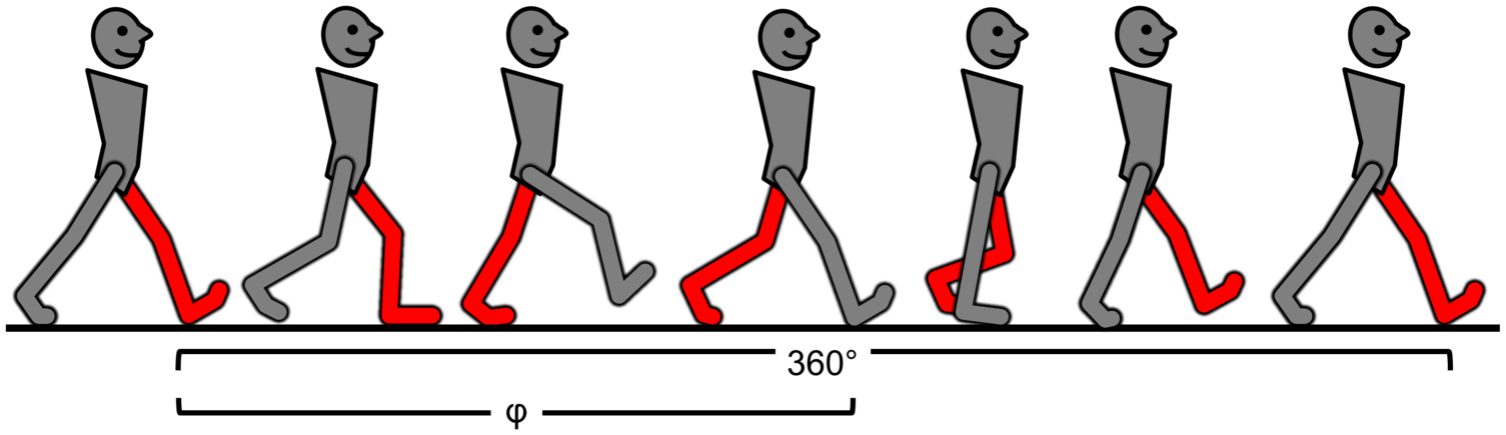
Left- right stepping phase definition, i.e., φ, with respect to the full gait cycle (360°). The time difference between consecutive heel strikes of the two feet (red and grey feet) normalized to gait cycle duration (red to red heel strike), is defined as the phase.

The PCI metric is based on a series of left- right stepping phases (φ_i_, ideally = 180°; [9]), briefly, the stride duration, i.e, the time difference between consecutive heel strikes of one foot is defined as a gait cycle (represented as 360°). The relative timing of contra-lateral heel strikes determines the phase, φ (Eq 1). i.e., φ is obtained by normalizing the step time with respect to the stride time. Ideally, φ = 180° for every step (Fig 1).

$$\varphi_{i}[º]=360{}^{\circ}\frac{t_{Ai}-t_{Bi}}{t_{B(i+1)}-t_{Bi}}=360{}^{\circ}\frac{step\;time\;A}{stride\;time\;B}$$ (1)

where t_Ai_ and t_Bi_ denote the time of the i^th^ heel strike of one leg and the other leg, respectively. It is noted that t_B(i + 1)_ > t_Ai_ > t_Bi_. The legs can be arbitrarily chosen, i.e. right as leg A and left as leg B or vice versa. Based on this, φ can be calculated as right step time relative to the left stride time (RreL) and/or left step time relative to the right stride time (LreR).

A vector series of φ_i_ is obtained for each gait trial. The PCI, expressed in percentile units, is the sum of the coefficient of variation of φ and the mean absolute difference between φ and 180°, normalized to 180° (Eq 2) [9].

$$PCI\;[\%]=\varphi_{\_CV}+P\varphi_{\_ABS}=100∙\frac{stdev(\varphi )}{mean(\varphi )}+100∙\frac{mean(\left|\varphi -180{}^{\circ}\right|)}{180{}^{\circ}}$$ (2)

### Data sets

The algorithm presented was developed based on training data which was simulated as described below. The results shown later demonstrate the performance of the algorithm on both real participants (young and elderly healthy, total n = 59) and on another set of simulated data (n = 20). These latter five data sets are considered as the test data sets.

All processing development and analysis of data was done via Matlab software 2015b (MathWorks, Inc.).

#### Simulation sets

The simulation generated a series of left-right stepping phase values (φ_*i*_) with 50 samples per ‘simulated participant’. From these a series of PCI values was obtained, where PCI_*k*_ is calculated based on a series of *k* φ values (*k*≠*1*; φ_*1*_, φ_*2*_… φ_*k*_… φ_*i*_). The data meets these two constraints: (a) each **φ** vector has a mean within the normative range, and (b) each ‘simulated participant’ produced final PCI values (i.e., based on all 50 φ values) compatible with those seen in young healthy adults [9]. To simulate the complementary stepping phases (i.e., for both cases RreL and LreR, see PCI definition above), stride times for one leg with mean stride time of 1.07±0.02 seconds [1, 37–40] were computed. Step times of the other leg were derived from Eq 1 and initializing the first HS at time 0 to obtain all HS event times.

**Fig 2:**
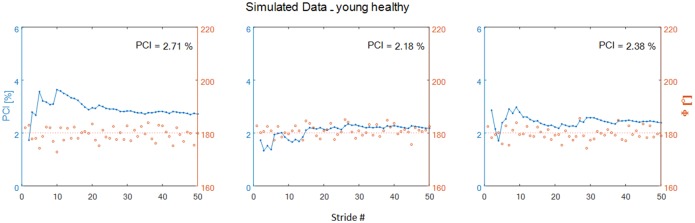
Examples of simulated data for 3 ‘virtual participants’. Generated φ values are marked by red circles (right ordinate) which are scattered, as expected in normal walking, around a red dashed 180° reference line. Calculated PCI values as a function of the number of strides is indicated by the blue circles connected by thin blue line (left ordinate). In the top right corner of each panel the calculated value of PCI for the complete set of the generated φ values (n = 50) is shown. It is noted that the data series are displayed as RreL (i.e: φ was calculated as Right leg step versus Left leg stride), the LreR values were also calculated.

Two φ vectors were obtained. From each φ vector a series of N-1 PCI values were calculated (*introduction* Eq 2) for *i* = 2,3,4…N gait cycles per 'virtual participant'. A few examples of the simulated data, i.e., φ vector and PCI values, are shown (Fig 2). It can be seen that in these examples the value of PCI fluctuates when only few strides are taken, and stabilizes when sufficient number of strides are taken. Simulated values of φ and PCI are available in the supporting files (S1 and S2 Files).

#### Physiological data

The experimental protocol was approved by the Human Studies Committee of the Sheba Medical Center and all participants provided informed written consent prior to entering the trials. For the present analyses we used four sets of physiological data:

1. Historical data [41] of 16 healthy young participants (self-paced treadmill -SPTM trials- see below; Age: 32.9 ± 5.5 y; 8 women).
2. Data from over ground (OG) walking—healthy young participants (n = 15; Age: 27.4± 3.2 y, 6 women).
3. Data from healthy elderly participants (SPTM trials; n = 15; Age: 68.7± 6.1 y, 5 women).
4. Data from OG walking—healthy elderly participants (n = 13; Age: 70.2± 5.5 y, 7 women).

Participants were included if they: (1) were between 20–40 years old (young) or ≥ 64 years old (elderly); (2) self-declared ability to participate in gait trials for about an hour. The exclusion criteria were: (i) any orthopedic, rheumatic, neurological or cardiovascular diseases, or morbidity in the past or present that affects joint range of motion, muscle strength, gait or balance; and (ii) inability to understand written or oral instructions.

### Apparatus and data collection and handling

#### SPTM protocol

**Fig 3:**
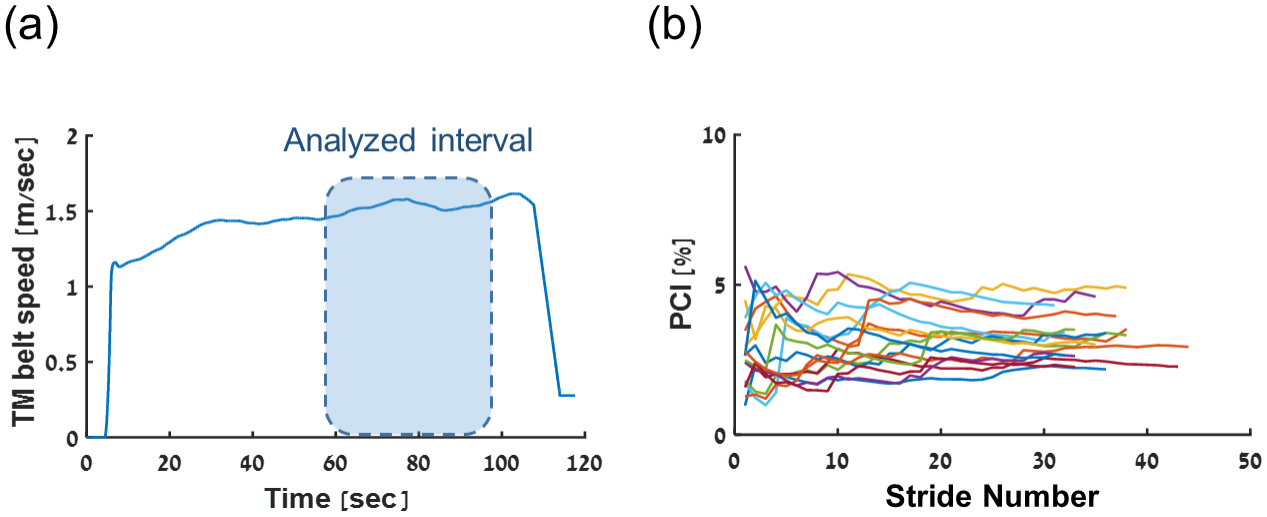
SPTM data collecting. (a) Treadmill belt speed graph of one participant walking in a self-paced comfortable walking speed. The shaded area, is the interval analyzed. (b) PCI data series of n = 16 young healthy participants.

A TM (R-Mill, ForceLink, The Netherlands) that is part of a virtual reality facility (V-Gait, Motek Medical, the Netherlands) was used. The TM speed was regulated in a self-paced mode by a built-in controller algorithm that is described elsewhere [41–43]. Thus, each participant walked at his/her own natural speed. Three-dimensional kinematic data of body fixed markers were recorded at 120 Hz with a VICON capture system (Oxford, UK); for the present analysis only heel marker data were used. Data from an interval characterized by steady state speed were analyzed in this study (see Fig 3).

#### OG protocol

Wireless Opal inertial sensors (APDM, Inc., Portland, OR, USA) were placed on the participant (at the lumbar spine and lower limbs). The Opal sensor includes triaxial accelerometers, gyroscopes and magnetometers and records signal data at 128 Hz. Each participant performed four consecutive 10 meter walking test (10MWT). The participants were asked to walk continuously back and forth, at a comfortable speed in a 24 m long corridor. Data from an interval characterized by steady walking without acceleration/deceleration/turns were analyzed in this study.

#### Heel strike detection

SP-TM protocol—A MATLAB GUI was built in order to assess gait cycle parameters, including heel strike (HS) (see section A in S1 Appendix for more information). HS events for this study were defined when the heel marker's vertical position was at minimum.

OG protocol—HS event times were obtained from the sensors placed on lower limbs. These event times were processed and analyzed via Mobility Lab software (APDM, Inc., Portland, OR, USA).

From these HS event times, the φ values were computed, and PCI data series were obtained for all participants. Data from these participants are included in the supporting information files (S3 File).

### Algorithm development

The equations for the phase φ and PCI were presented above (Methods Eqs 1 and 2). Since both the elements comprising the PCI metric (i.e.: φ_CV; Pφ_ABS) are based on means from accumulative data from {1, 2, …, n,…N-1} strides, it is expected that as n grows PCI stabilizes, provided walking conditions are not changed. However, until reaching this stabilization, different patterns of fluctuations may appear in the PCI values. Therefore, a definition of a point of stabilization (POS) suitable for all types of fluctuation patterns is needed.

Herein an algorithm is proposed to define the POS, i.e., the required number of strides needed to provide a reliable PCI value estimation which represents reaching steady state (i.e., from this point, all calculated PCI values should be similar).

*Input*: A vector of N-1 consecutive PCI values. The n^th^ value in this vector is PCI calculated based on n-1 gait cycles.

*Output*: POS.

Rationale for the proposed algorithm:

A CV-based algorithm was developed. The algorithm utilizes a moving window that analyzes the data over a predefined interval of K consecutive values and computes the CV of the PCI values within the window. A criterion of *CV* ≤ 0.05 was set to establish that PCI is stable over a given block (see section B in S1 Appendix for validation of initial block size). Note that the CV threshold assesses the fluctuation of the PCI signal, independent of whether the PCI has high values (i.e., less coordination) or low values (i.e., more coordination, e.g., in young healthy).

The algorithm operates in two stages: (i) systematic search for the Region of Interest (RoI), and (ii) Localization of the POS.

#### Stage 1- Systematic ‘backward’ search for RoI (Fig 4a–4c)

**Fig 4:**
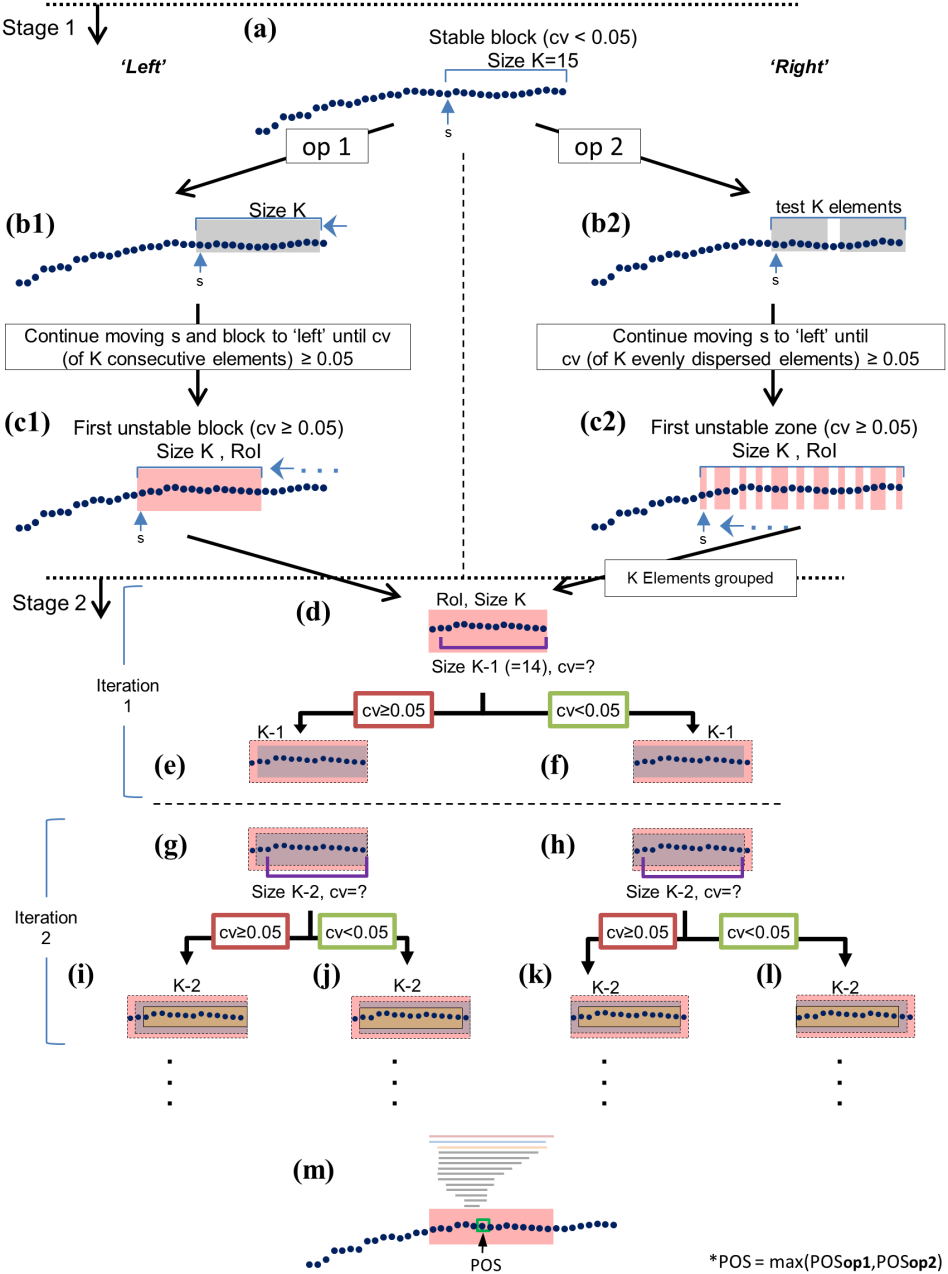
POS determining algorithm illustration. Stage 1: Gross location of the Region of Instability (RoI) (a-c). (a). the rightmost block of K = 15 is checked for stability starting from s to the ‘right’. Two options (op 1 and op 2), both applied in the algorithm, are performed if stable (CV≤0.05) → (b1). Addressing the next block of K elements, by moving this window to the left / (b2). Moving s to the left, testing K = 15 evenly dispersed elements → (c1). Repeated, constantly moving the window, until the first unstable block of 15 elements is found, and defined as the Region of interest (RoI) / (c2). Repeat moving s to the left, and testing K = 15 elements dispersed, until stability not met, define these elements as the RoI. RoI is shaded in red. Stage 2 is the "fine-tuning" step (Fig 4d-m). An iterative process begins to find the POS. Each iteration results in choosing the right or left sub-block of size K-1. (d) first, the algorithm tries to omit the leftmost point in the RoI and checks stability. (f) if the criterion of stability is met, the left sub-block of the same size is chosen for further testing. (e) if the criterion of stability is not met the right sub-block is chosen. (g-l) Process continues with size of K-2. The algorithm stops when K = 2, and returns the index to the right element of the unstable window as the POS. (m) illustrates the whole process and shows the unstable blocks and the resulted POS (shown via a green square).

The purpose of this iteration process stage is to find (from ‘right’ to ‘left’, index moving leftward defined as S) the first window of PCI values that are "unstable”, i.e., the RoI. Instability is defined if either one of the following happens: (1) The K = 15 consecutive values starting from S to the ‘right’ have CV>0.05 (Fig 4a, 4b1 and 4c1); (2) The 15 values evenly dispersed from S to the last stride (‘rightmost’) have CV>0.05 (Fig 4a, 4b2 and 4c2). Once S is found, the respective 15 elements (block of K = 15) from option 1 and 2 (please note that in option 2 the 15 dispersed values are now “compressed” to generate the block, see passage from c2 to d) are passed on to stage 2 and are defined as the RoI (where stability isn’t met).

For example, suppose the PCI data series consists of 50 values. The instability check for the S = 21^st^ stride will involve CV computations for (1) strides 21,21,23,…,35 and (2) 21,23,25,…,50. If both CV are below 0.05, the algorithm moves on to check the 20^th^ stride. Otherwise, the 15 corresponding values of PCI are passed on to stage 2. The POS is determined in stage 2 for both options from stage 1, and the maximum between the two is set as the POS.

#### Stage 2- Localization of the POS within the RoI (Fig 4d–4m)

Once the RoI is identified, an iterative process begins to find the POS. This stage acts as a higher resolution "fine-tuning" step that tests stability of smaller windows within the RoI, while looking for the transition point between instability and stability. The algorithm keeps decreasing window size, and iterates on either left or right sub-windows until reaching a stopping condition of block size k = 2, and returns the element to the right as the POS, which is the onset of stabilization. In other words, the algorithm increases its resolution in order to 'zoom' into this unstable block, and searches again for the unstable sub-block within.

See an illustrative example (Fig 4); the figure legend that shows all the stages. A flowchart also available in the supporting files (S1 Fig).

#### Estimation of the algorithm performances

The POS was obtained for each participant. The estimated required number of strides to reach POS was obtained by the group mean and defined as *mean POS*. Estimated required number of strides to reach POS for RreL, LreR and average PCI (i.e., 3 groups) was compared by paired *t*-tests for the simulation test set. Algorithm performance was assessed by evaluating the true error (TE) and absolute error (AE) between the PCI value calculated based on the mean POS estimated strides, versus the overall PCI value calculated from all strides. These are calculated via Eqs 3 and 4 respectively.

$$TE=\frac{PCI(mean\;POS)-PCI(end\;value)}{PCI(end\;value)}$$ (3)

AE=|TE| (4)

The distributions of TEs were plotted in a histogram and tested with the Shapiro–Wilk normality test. The distributions were assumed to be approximately normal (W = 1 for the normal distribution). If the test rejected normality (p < 0.05), normal quantile–quantile plots would need to be examined to determine the nature of the deviation from normality [44].

### Statistical analysis of physiological results

For each data set, mean and SD of the POS detected by the algorithm was computed. One-way ANOVA test was used to determine differences between the means. If the null hypothesis was rejected (significance level of 5%), post hoc tests were run to compare two groups.

## Results

### Simulated set results

Applying the algorithm on the PCI data series, we found that the mean number of strides needed to reach POS across all 20 simulated data sets, for PCI RreL, PCI LreR and average PCI, was 22.4 ± 9.4, 20.2 ± 8.4 and 19.4 ± 8.4, respectively (p>0.2; for all possible within-groups comparisons, paired t-test). POS data for each ‘simulated participant’ is available in the supporting files (S4 File). This algorithm was also compared to two other methods. See section C in S1 Appendix.

**Fig 5:**
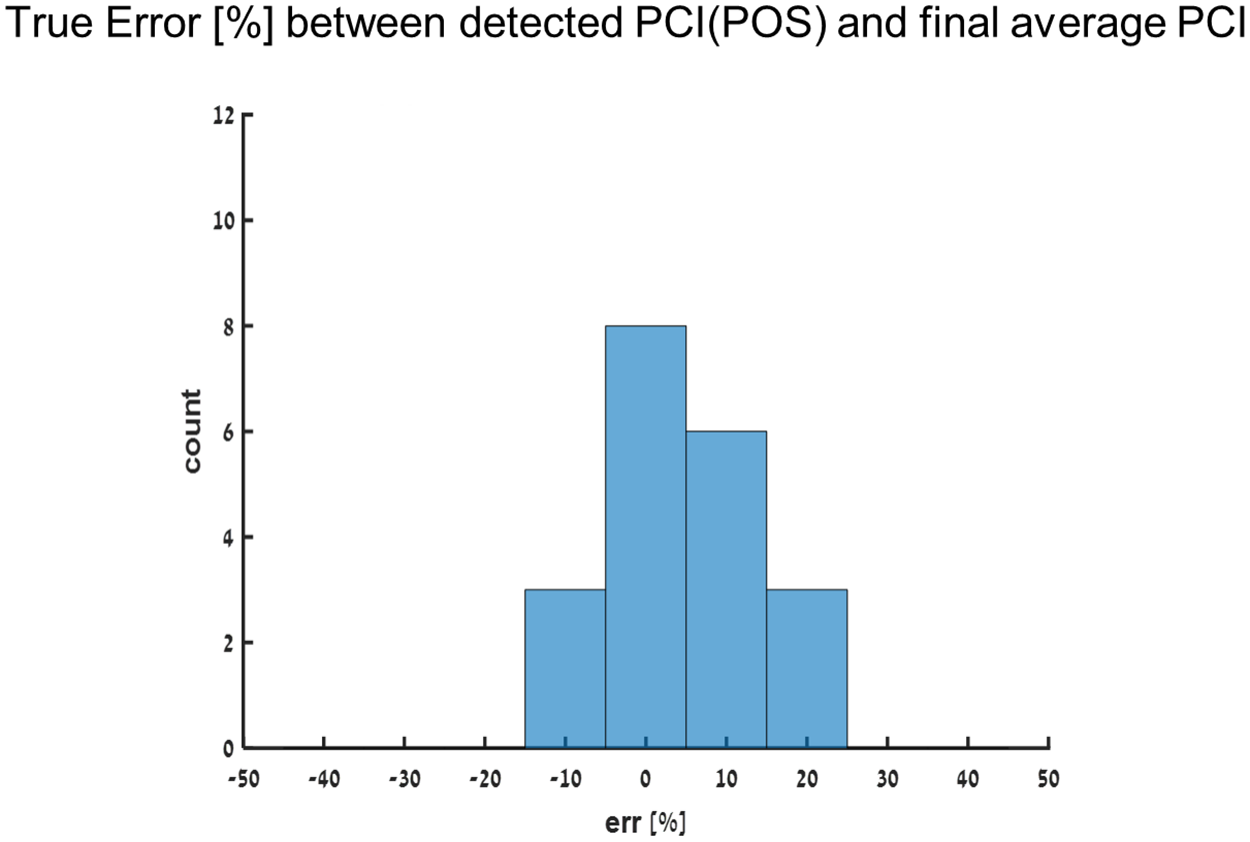
True error histogram. Histogram with bin size of 10% summarizing the true error percentage % between the POS value of the PCI vs. the last PCI value in the series (n = 20). Shown for average PCI.

Based on these results we set 23 strides as *mean* POS = 23, the required number of strides to obtain a reliable estimate of the PCI for a stabilization criterion of 0.05. The true error (TE) between the 23^rd^ value and the end value was calculated for each ‘simulated participant’. The distributions of the TE of the comparison of PCI values are presented in a histogram (Fig 5), and it closely approximates that of a normal distribution (W > 0.94, p-value > 0.05). The average AE for the characteristic PCI from all ‘simulated participants’ is 7.05 ± 6.07%.

### Physiological results

**Fig 6:**
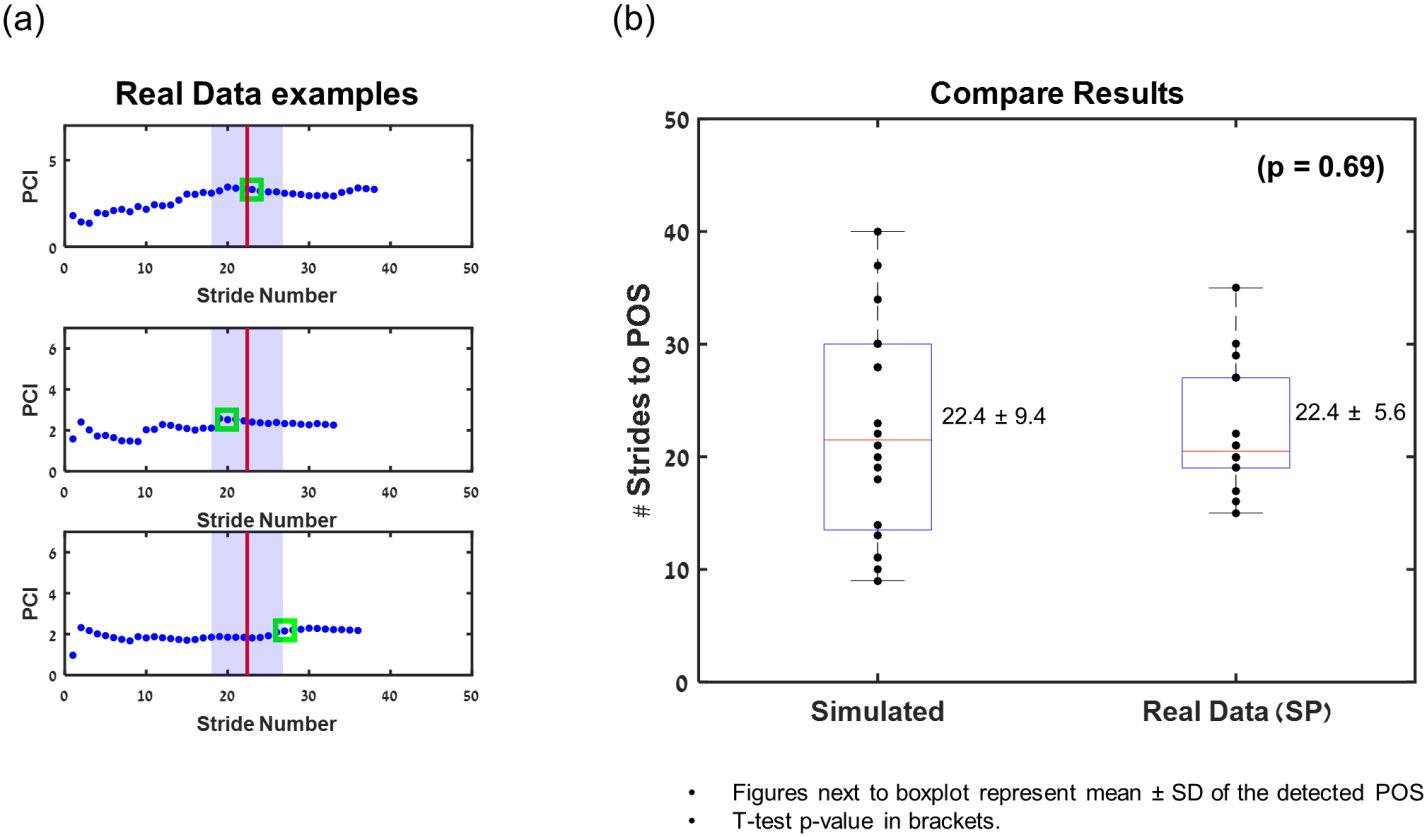
Comparison physiological set vs. simulated set. (a) Detection examples for three young healthy real participants. A green square marks the detected POS; a red line and shaded area represent the mean and 95% confidence interval for the mean number of stride to reach POS obtained from ‘simulations’. (b) Comparison between POS values obtained from simulated vs. physiological data (group A).

Fig 6 depicts application of the algorithm to real data (Fig 6a), and provides comparison of detected POS values from 16 real data sets (Group A) to simulated data sets (n = 20; Fig 6b).

Table 1 summarizes the results of the algorithm performance per each group (A-D), i.e., the mean POS values for RreL data sets. PCI values and walking speed are also provided. There were no statistically significant differences between group means as determined by the one-way ANOVA (F(3,55) = 0.92, p = 0.44). The cross group mean value of POS was 22.6 strides (SD = 8.6). Similar results were obtained in a post-hoc analysis adding the simulated data (see above) as a fifth ‘group’ (F(4,74) = 0.65, p = 0.63).

**Table 1:** Summary of *mean POS* per each data set of real participants, walking at a comfortable speed.

	(Group A) Young SPTM (n = 16)	(Group B) Young OG (n = 15)	(Group C) Old SPTM (n = 15)	(Group D) Old OG (n = 13)
PCI (%)	3.3 ± 0.83	3.4 ± 1.1	4.4 ± 1.4	5.03 ± 2.07
POS (#strides)	22.3 ± 5.6	24.9 ± 12	22.4 ± 5.5	19.6 ± 9
AE (%)	6.3 ± 4.4	11.1 ± 8.2	6.2 ± 5.4	8.9 ± 5
TE (%)	-2.7 ± 7.3	-0.5 ± 14.1	0.5 ± 8.4	4.2 ± 9.5
Gait speed (m/sec)	1.4 ± 0.2	1.3 ± 0.16	0.99 ± 0.34	1.2 ± 0.15

SPTM, self-paced treadmill walking; OG, over ground walking; AE, Average error; TE, true error. Numbers shown are mean±SD.

The correlation analyses showed no significant correlation between gait speed values and POS values (Group A: spearman’s ρ = 0.12, p = 0.66; Group B: spearman’s ρ = 0.21, p = 0.46; Group C: spearman’s ρ = -0.11, p = 0.68; Group D: spearman’s ρ = -0.15, p = 0.63), suggesting that algorithm performance is unrelated to gait speed.

**Fig 7:**
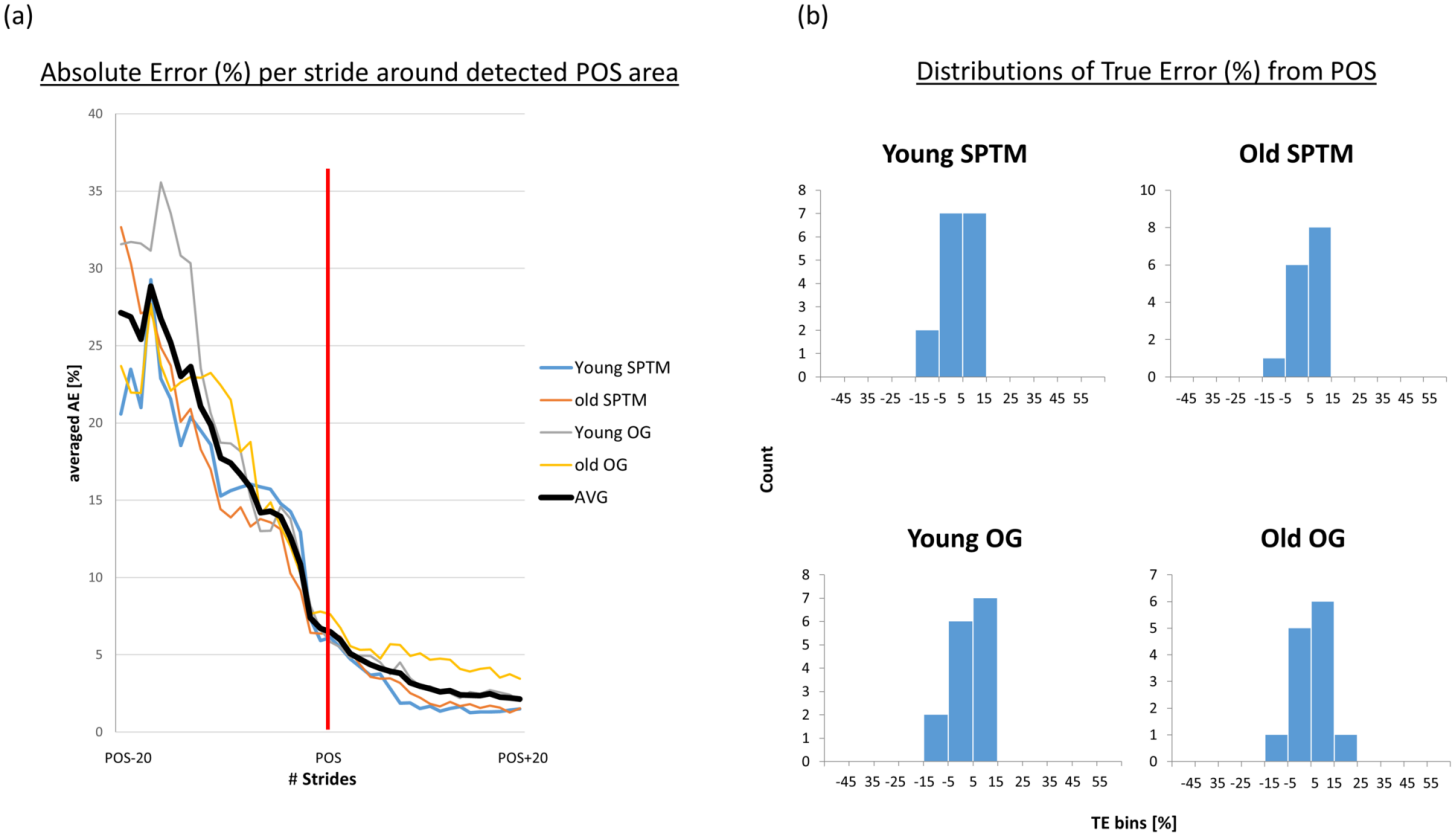
Algorithm performance on real data sets. (a) The average of the last 3 PCI values was considered the ‘final PCI’. PCI data of all participants were aligned to POS. The absolute error from the i^th^ stride after POS was calculated for POS+i, where i = {-20,…,20} and averaged per group. Note that each of the four lines shows the average of one tested group of participants. As expected, the error significantly decreases before POS and only slightly decreases after POS. It is noted that the PCI vector was padded with the first and last PCI values at either end to obtain a larger vector in cases where there are less than 20 values before/after POS per participant in each group. (b) Distribution of the True Errors calculated from the detected POS relative to the ‘final PCI’.

**Fig 8:**
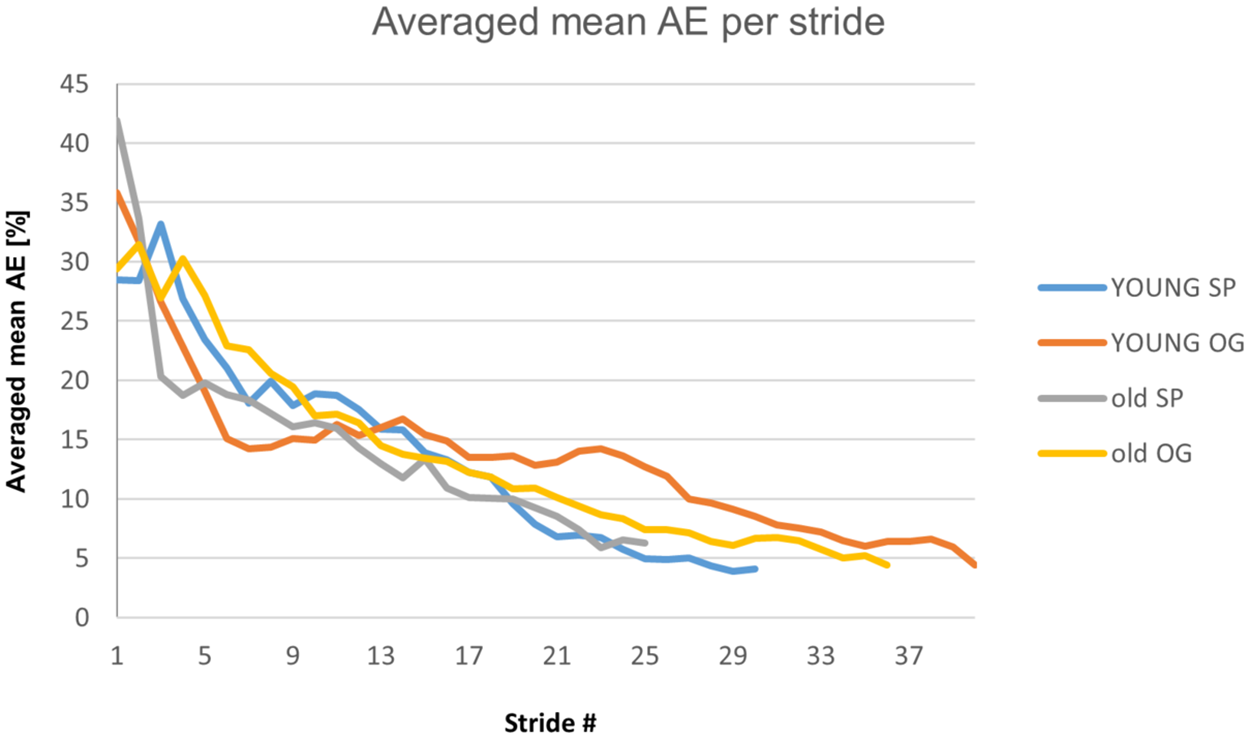
Plots illustrating the absolute error per stride (independent of the algorithm) averaged per participant within each group.

With respect to real gait data, algorithm performance is illustrated in Fig 7, where the AE drops drastically before the POS detection and decreases moderately after that. Fig 8 and Table 1 summarizes results per group. TE and AE between the mean POS PCI value and the final value was calculated for each participant. The distribution of the TEs are close to that of a normal distribution (W > 0.9, p-value > 0.05). These results are also shown in Table 1. POS detection per participant can be found in the supporting information files (S4 File)

## Discussion

A newly developed algorithm, that estimates the required number of gait cycles for assessing PCI values, was applied on heel strike timing data. Our findings in multiple datasets support the conclusion that about 23 gait cycles, on average, are sufficient to reliably define a participant’s characteristic PCI value. The algorithm is based on the use of a steady-state detection algorithm based on size-adapting moving windows, and a stability criterion based on the CV of the values within the window.

### Point of stabilization of gait parameters—Earlier estimations from the literature

Lord et al. [45] reported uncertainty about the number of steps required for optimal assessments of gait variability, but suggested collecting at a minimal number of 12 steps, based on intraclass correlation coefficients (ICCs) for Test-Retest Reliability.

Hollman et al. used a pressure sensitive sensor carpet (i.e., GAITRite) for recording intermittent walking trials. They measured velocity, cadence and variability in stride velocity. The number of strides required to achieve reliability was estimated by the Spearman-Brown prophecy formula. They concluded that about 370 strides are recommended for gait variability estimation [2]. Galna et al concluded that gait variability was more reliable during continuous walks (rather than intermittent protocol), and stated that 50 steps was the optimal number of steps needed for reliable estimation [46], a figure which is comparable to the results of the present work (i.e., ~23–25 strides = ~46–50 steps).

As the aforementioned estimations were obtained from repeated trials and theoretical formulas (e.g., ICC & Spearman-Brown prophecy [2]), an automated detection algorithm for real data like the one proposed here is preferable as it 'interacts' with the actual collected data making only one a priori assumption (i.e., more gait cycles lead to a more stable value).

### Advantages of the method presented in this article

Compared to other existing steady state detection methods (see Introduction), the CV-based algorithm presented here has the advantage of an adaptive size, whereas other methods using a window can detect an area and not a single point. Other disadvantages of existing methods may include: complexity, large calculations, model-fitting, offline application only, need to adjust parameters arbitrarily, etc.

The following are advantageous elements that can also be noted: (a) the algorithm is based on a well-known and easy-to-understand statistic measurement (i.e., CV); (b) it is insensitive to the absolute values (e.g., of the PCI), and considers only how they relate to each other; this enables setting one threshold criteria (in the present study, CV = 5%) for various data sets; (c) The algorithm can be easily adjusted to be more or less rigorous, via a single parameter (i.e., threshold for the CV criterion); (d) it also mimics human action—low-resolution examination followed by a high-resolution fine tuning step; (e) the window decreases in size in correspondence to the variance of the data, automatically adjusting to the data; (f) the CV metric enables the algorithm to detect stability while considering not only the variance of the signal, but also how this variance is compared to the absolute values of the overall signal. This is useful where the higher the value of the signal, the higher the tolerance for its variance (e.g., a signal oscillating between the values 99–101 is considered “more steady” (1% change in signal) than a signal oscillating around values 1–3 (>1% change in signal). Standard methods like linear regression SDM do not take this tolerance into account; (g) the algorithm is applied on data from a single walking trial and returns a POS, i.e., no need for repeated trials as in the ICC method [2, 46].

### Implications

Several implications and future directions arise from the present work. We introduced a generic method to assess the minimal number of gait cycles in order to obtain a reliable measure that is based on parameter's statistics. It was demonstrated here on the PCI metric but we posit that it can be generalized to other measures.

It is important to note that from practical point of view, the method provides those who are designing gait experiments additional degrees of freedom in protocol planning.

For example, we found that on average (based on simulated data and 59 empirical examples) PCI values stabilizes on average after 22.6 strides (SD = 8.6). It is therefore up to the experimenter to decide upon the margins he/she will apply (e.g., setting a minimum of mean + x*SD strides).

As the algorithm operates on data from a single walking trial and returns a POS, it may be beneficial for several applications. For example, in the future, this algorithm can be incorporated within the recording device that obtains all the data in real time, as it only takes into account ‘previous’ data, and indicates whether more steps must be recorded to achieve the stabilized PCI estimate.

### Study limitations and future directions

Some limitations of this study should be acknowledged. Our pilot analyses [47] suggests that this estimation is compatible with real gait data, yet this should be further established.

This algorithm also addresses some edge cases where there is a slow and moderate uniform slope in the data which has a CV which is below the threshold (i.e., branch of op2 is applied). Such an instance may introduce a systematic error of ±1–2 strides in the detected POS.

The described algorithm to ascertain the minimal strides required for reliable estimation of PCI was assessed on gait segments that were derived from steady state walking velocity periods obtained from trials performed with a self-pace treadmill. Indeed, in self-paced treadmills considerable walking is carried out before gait speed stabilizes (e.g., ~37 meters see [41]), however, during over ground walking only 2–3 (~2 meters) strides are required to establish a constant walking speed (10MWT). Thus practically, gait cycle data should be evaluated with respect to the procedure in use.

Finally, to demonstrate efficacious usability analyses employing the present algorithm should be applied to other gait metrics calculated based on parameters' statistics (e.g., stride-to-stride time CV, see preliminary outcome in section D in S1 Appendix) and to participants with gait impairments.

## Supporting information

S1 File Simulated phi values. (XLSX)

S2 File Simulated PCI values LreR, RreL and average PCI vectors. (XLSX)

S3 File Real PCI vectors for all physiological data sets. (XLSX)

S4 File POS values of ‘simulated participants’ and physiological participants. (XLSX)

S1 Fig Flow chart depicting the 2-stage process of the POS-determining algorithm (Op1). Terminology used for variable naming: window_size—size of the current moving window, i- index of the elements in the PCI data iϵ{1,2,….,N}, the process starts with i = N, vec—the current segment of PCI values to analyze (length of vec matches window_size). CV—coefficient of variation. POS—point of stabilization. The process starts with an input of a vector of N consecutive PCI values, the algorithm analyzes the CV of sliding windows, and decreases window size until the POS is determined (Op 1 is depicted in this flowchart. For the whole process see Fig 4 in manuscript).
(TIF)

S1 Appendix Appendix file for further information. (PDF)
