# Supplementary figures and images for: How many strides are required for a reliable estimation of temporal gait parameters? Implementation of a new algorithm on the phase coordination index
Source: PLoS One. 2018 Feb 8;13(2):e0192049. doi: 10.1371/journal.pone.0192049 (PMC5805232; doi:10.1371/journal.pone.0192049)

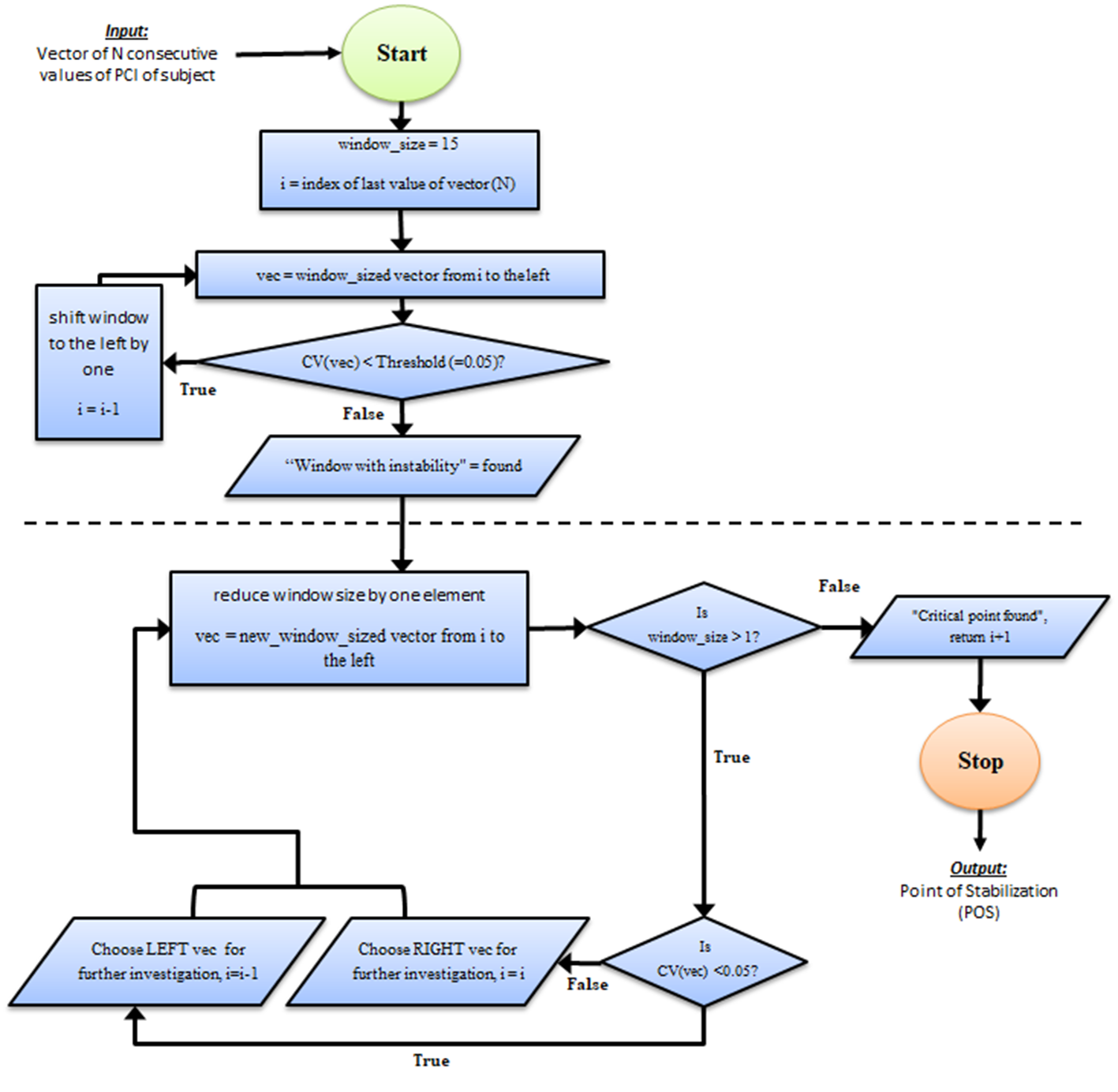

Supplement: S1 Fig — Terminology used for variable naming: window_size—size of the current moving window, i- index of the elements in the PCI data iϵ{1,2,….,N}, the process starts with i = N, vec—the current segment of PCI values to analyze (length of vec matches window_size). CV—coefficient of variation. POS—point of stabilization. The process starts with an input of a vector of N consecutive PCI values, the algorithm analyzes the CV of sliding windows, and decreases window size until the POS is determined (Op 1 is depicted in this flowchart. For the whole process see Fig 4 in manuscript). (TIF) [file pone.0192049.s005.tif]
