# Supplementary material for: How many strides are required for a reliable estimation of temporal gait parameters? Implementation of a new algorithm on the phase coordination index
Source: PLoS One. 2018 Feb 8;13(2):e0192049. doi: 10.1371/journal.pone.0192049 (PMC5805232; doi:10.1371/journal.pone.0192049)
Supplement: S1 Appendix — (PDF) [file pone.0192049.s006.pdf]

S1 Appendix:

A. Validation of Heel-Strike detection

Participants walked on a split-belt treadmill that was equipped with force plates for each belt. These forces were recorded at 120 Hz, filtered with a fourth-order, zero-lag, Butterworth filter with cutoff frequency using the residual method of Wells and Winter [1] .

Generally, force plate HS detection is considered a gold standard. HS was considered to occur when the vertical force crossed a threshold of 20N [2]. For this paper FP data was not usable due to participant leg-crossing from one belt to the other (Fig A). A validation process comparing HS events from force plate versus kinematic HS events had to be conducted.

HS, using the kinematic data, was defined when the vertical position of the heel marker was at minimum, with minimum distance of 80 samples between these points. The validation process was run only at certain time intervals where there was no leg-crossing.

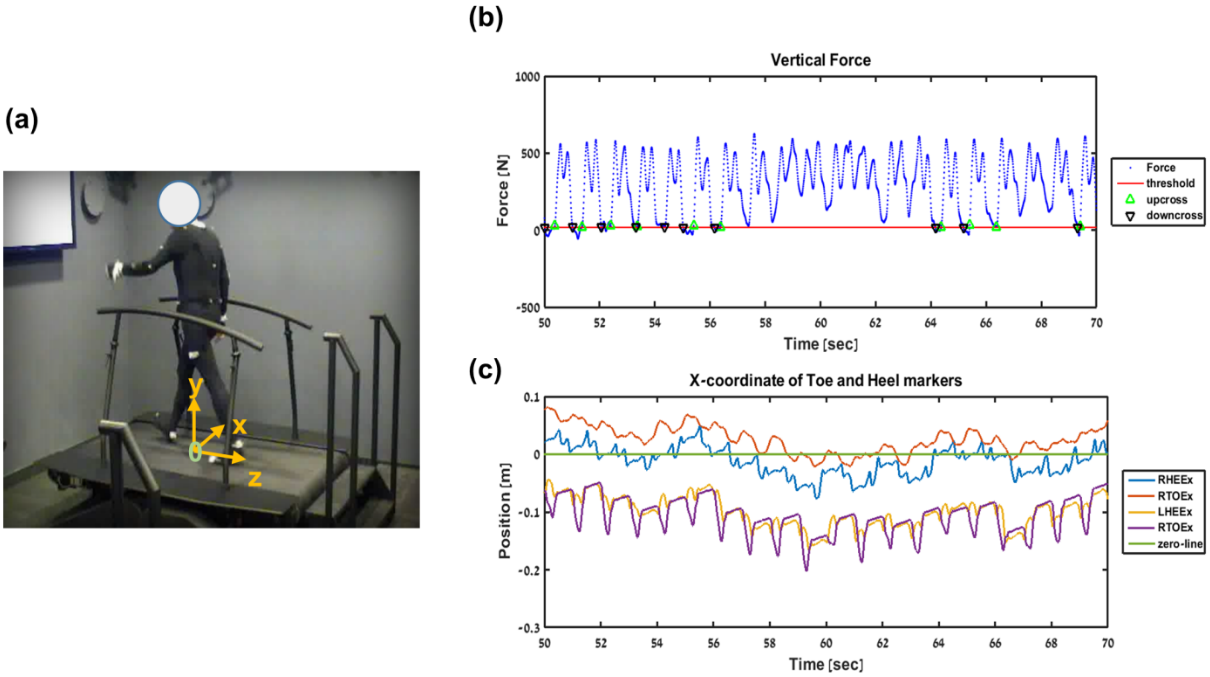

Fig A. Leg-crossing during treadmill walking. (a) Participant walking on TM with axes origin shown. (b) Vertical force graph with the threshold line of 20N. (c) x coordinate of the feet markers. When both feet are on the same force plate, the forces rise above threshold making it hard to detect heel strike timing.

Data were processed offline in MATLAB™ 2015b software (MathWorks, Inc.). The true timing errors (TTE) and absolute timing errors (ATE) were calculated for each trial using:

(5) $TTE = t(HS_{kinematic}) - t(HS_{FP})$

(6) $ATE = |TTE|$

Taking into account only relevant intervals for the validation process resulted in an average TTE of 0.009 ± 0.03 sec mismatch between HS detection from force plate and vertical heel marker position. The average ATE is 0.023 ± 0.02 sec for HS. These values are shown in the following table. This average is concurrent with the offset errors reported in previous work [2-4]. This validation process was implemented on 5 randomly chosen participants.

Table. Timing error calculations per participant.

Participant	True Timing Error HS mean±SD (sec)	Absolute Timing Error HS mean±SD (sec)
**1**	0.004±0.02	0.016±0.01
**2**	-0.006±0.02	0.019±0.006
**3**	0.029±0.03	0.029±0.03
**4**	-0.016±0.03	0.016±0.03
**5**	0.034±0.02	0.034±0.02
**Total 5-participant mean**	**0.0099±0.03**	**0.023±0.02**

B. Validation of initial block size

In order to assess the impact of block size used for the evaluation of the stability point (block size=15, cv Threshold=0.05), the same algorithm was applied for various block sizes. The impact on the resulted point of stability can be seen in Fig B. It is apparent that choosing any initial block size of 13 or more results in a similar point of stability. Choosing a smaller block size leads to variance in the results, a direct consequence of the small sample size used in the first step of the algorithm. Therefore, an initial block size of 15 was chosen in this article.

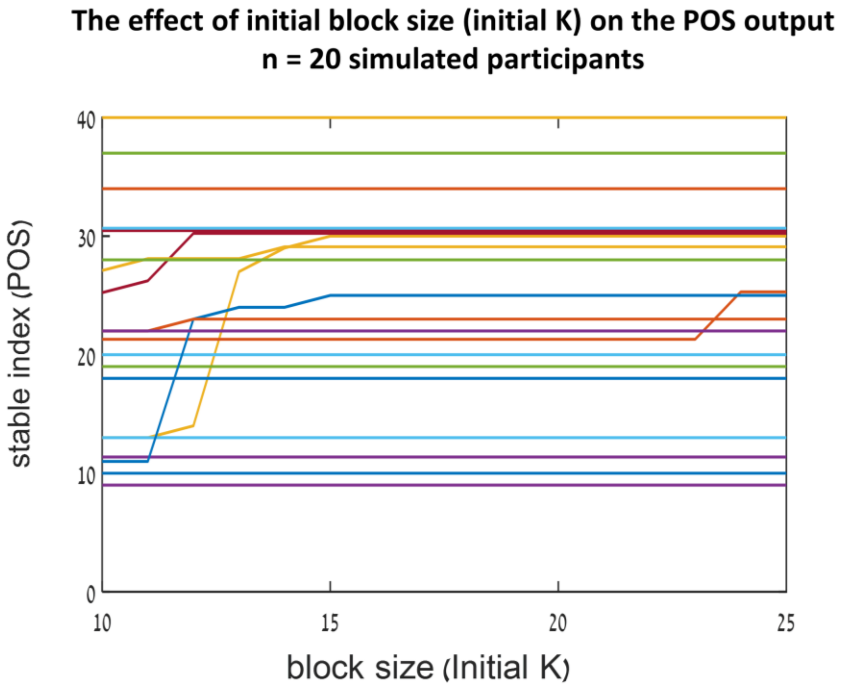

Fig B. **Effect of block size on POS per 'simulated participant'**. Output of stable index remains the same after certain block size. A block size of 15 was then chosen for the algorithm.

C. Comparison to two other algorithms

Two methods were chosen from among widely used online steady state detection methods - SDM, in which slope (i.e., trend in the data) is addressed [5, 6] and the VRT method, in which the variance ratio is tested [7]. To maintain consistency among these methods, the window size was chosen to be 15 and the stability test direction was set from the end of the series to the beginning (i.e., from 'right' to 'left') shifting the window by 4 values to the left each time and performing the test again.

The SDM focuses on evaluating a regression line y = ax+b. A linear regression *t*-test is performed to determine whether the slope of the regression line is significantly different from zero. If the slope of the regression line is significantly different from zero, it is concluded that there is a significant relationship between the independent and dependent variables, thus not reaching steady state yet. If the null hypothesis is not rejected it is assumed that the slope is zero. If the slope of the window is considered zero 3 times consecutively it is assumed steady state is reached.

The VRT method is a test of ratios. The test statistic is the ratio between two kinds of variances. One is the mean squared deviation from average (v1) and the second is mean squared differences of successive data (v2). If the ratio v1/v2 is greater than 1, the window is not considered stable. If it is less than and proximal to 1, it is considered stable. As stated, this test is done for each window, and if a stable window is identified at least 3 consecutive times, it is assumed that a steady state has been reached.

Comparing the newly presented method to these methods is accomplished by plotting a Bland-Altman plot [8]. The first step is to plot the data and draw the line of equality (='unity line'). This plot is useful for visual assessment of agreement. However, it is preferable to plot the difference between the methods against their mean. Provided differences lie within mean ±2*SD (i.e., “limits of agreement”), we can use the two measurement methods interchangeably.

For the simulated data sets, we estimated the required number of gait cycles to reach a stable PCI value, using the SDM and VRT methods. A minimum of 24.5±7.1 and 24.0±7.9 gait cycles were estimated respectively and are thus consistent with the results from the proposed algorithm.

The average AE for the characteristic PCI from all ‘simulated participants’ between the 24th and the last PCI is 7.75 ± 6.67 %, similar to the AE value based on the new algorithm.

Fig C shows how these methods relate. As there are three methods to compare, three series are shown. Line y=x represents ideally matched methods. The data are then replotted as a Bland-Altman plot of the three series showing good agreement. As can be seen in the figure, the measurements lie within the “limit of agreement”, indicating each of the two methods that we compared are acceptable.

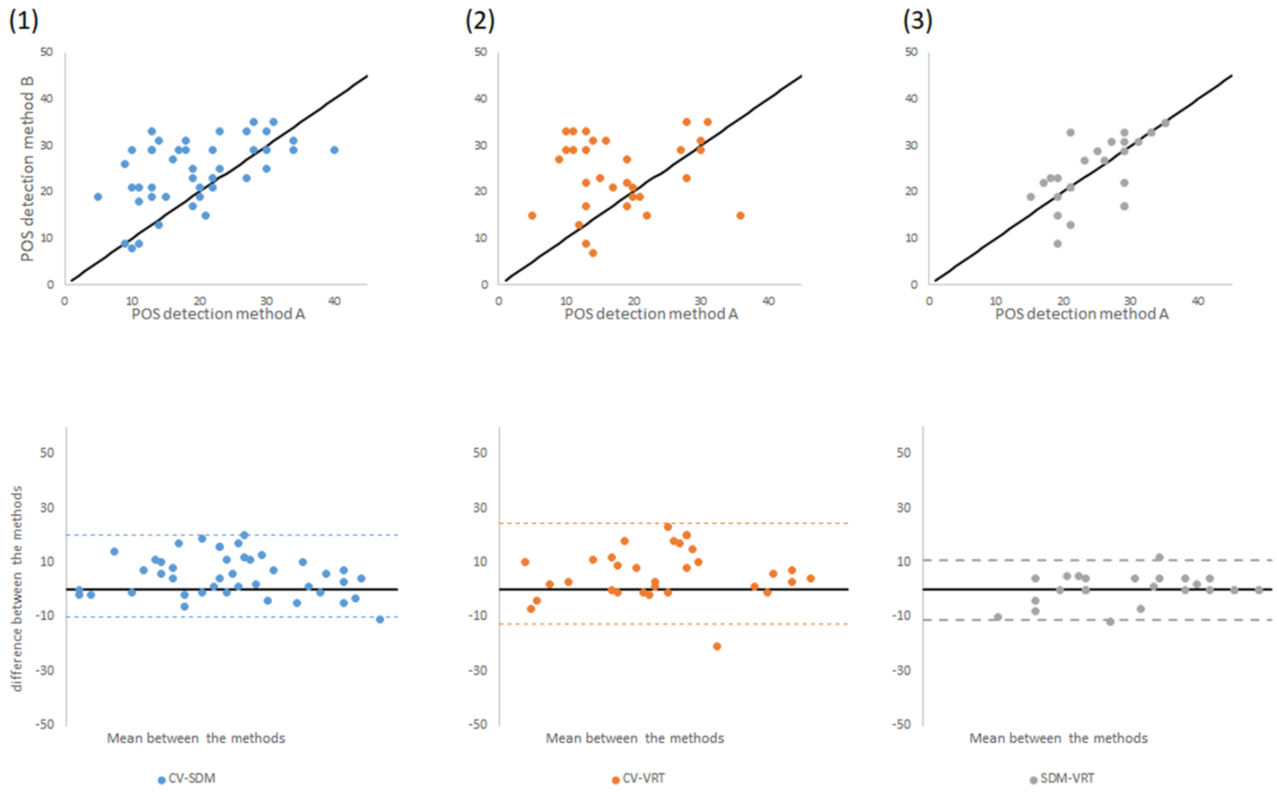

Fig C. Plots assessing agreement between pairs of methods. Three comparisons (blue, orange, gray) are shown (1) CV-SDM (left upper and lower panels); (2) CV-VRT (middle panels); (3) SDM-VRT (right panels). Method A (abscissa in the upper panels) corresponds the first indicated method of the pair, and Method B (ordinate upper panels) corresponds the second

indicated method (e.g., in the left panel Method A = CV, Method B = SDM). Each point represents the POS values of one ‘simulated participant’ obtained by the compared methods. Top panels: distributions relative to the unity line. Bottom panels - the same data replotted as a Bland-Altman plot. Dashed lines show the ‘limit of agreement’ between the methods (mean±2*SD). CV - method presented in this article; SDM-Slope Detection Method; VRT – Variance Ratio Test.

To evaluate the advantage of the present CV-based method in comparison to the other methods, examples of ‘participants’ with the lowest correlation were picked (i.e., far from the unity line and/or far from the zero-line in the Bland-Altman plot) for further inspection. Fig D depicts the results of this assessment.

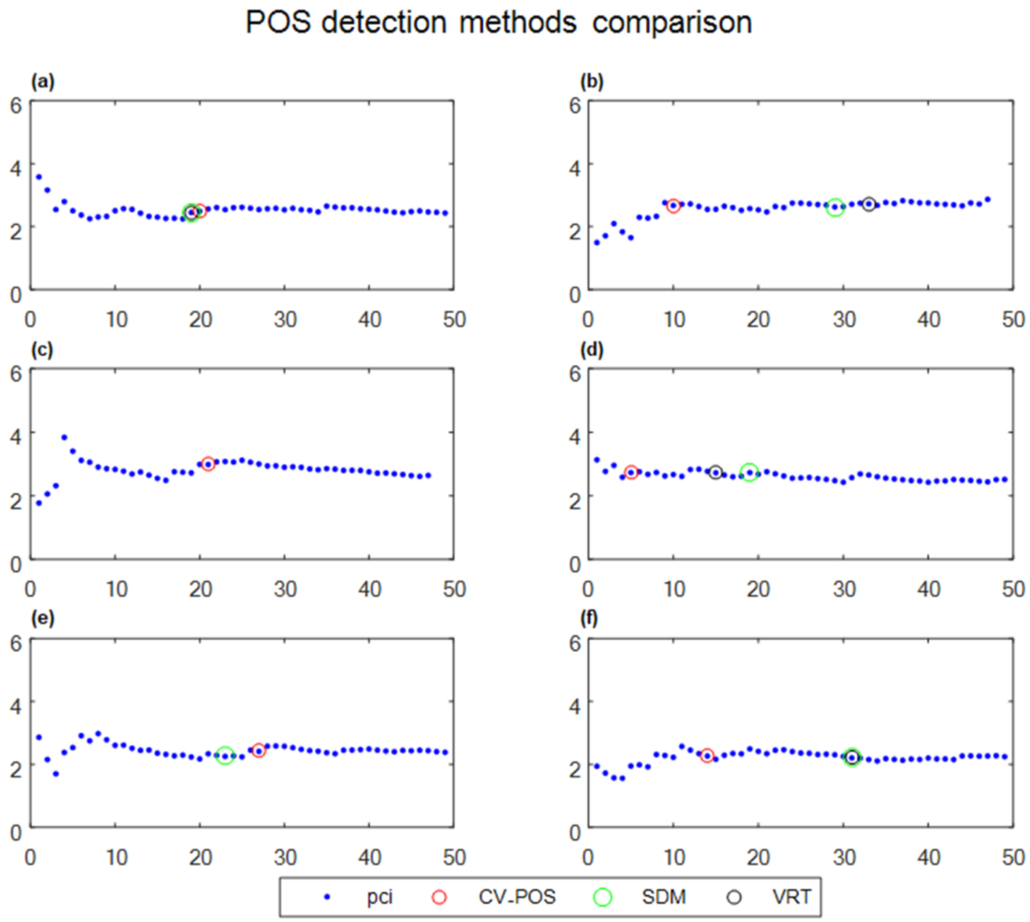

Fig D. “Mismatching cases” inspection. (a) Classical case in which all methods detect relatively similar POS values. It appears that CV-POS algorithm can: 1) find a POS, in cases that the other methods may not (c,e,g), and 2) agrees with visual inspection for the beginning of the stable period (b,e,f).

D. Feasibility of applicability to other gait parameters – strides required for stride-time-CV

This algorithm may potentially be applied on other cumulative gait parameters. The algorithm was applied on the stride-time-CV parameter computed from the young healthy SPTM data set (Group A; n=16). Stride-time-CV is defined as the coefficient of variation of cumulative stride times, where stride time is the time from one HS to the next for the same limb. The results for reaching POS for stride-CV-R (right limb) and stride-CV-L (left limb) were 20.7 ± 7.8 and 20.1 ± 8.7 respectively. The mean POS attained for the limbs are comparable (t-test, p>0.6).

References

1. Wells R, Winter D. Assessment of signal and noise in the kinematics of normal, pathological and sporting gaits. Human locomotion. 1980;1:92-3.
2. Hreljac A, Marshall RN. Algorithms to determine event timing during normal walking using kinematic data. Journal of biomechanics. 2000;33(6):783-6.
3. O'Connor CM, Thorpe SK, O'Malley MJ, Vaughan CL. Automatic detection of gait events using kinematic data. Gait & posture. 2007;25(3):469-74.
4. Zeni J, Richards J, Higginson J. Two simple methods for determining gait events during treadmill and overground walking using kinematic data. Gait & posture. 2008;27(4):710-4.
5. Bethea RM, Rhinehart RR. Applied engineering statistics: CRC Press; 1991.
6. Wu J, Zhou S, Li X. Acoustic emission monitoring for ultrasonic cavitation based dispersion process. Journal of Manufacturing Science and Engineering. 2013;135(3):031015.
7. Crow EL, Davis FA, Maxfield MW. Statistics manual: with examples taken from ordnance development: Courier Corporation; 1960.
8. Bland JM, Altman D. Statistical methods for assessing agreement between two methods of clinical measurement. The lancet. 1986;327(8476):307-10.
